# Stereological assessment of the effects of vitamin D deficiency on the rat testis

**DOI:** 10.1186/s12902-020-00642-0

**Published:** 2020-10-29

**Authors:** Ali Zamani, Forough Saki, Neda Hatami, Farhad Koohpeyma

**Affiliations:** 1 Endocrine and Metabolism Research Center, Shiraz University of Medical Sciences, Nemazee Hospital, Zand Avenue, Shiraz, 7193635899 Iran; 2 Division of Endocrinology and Metabolism, Department of Internal Medicine, Shiraz University of Medical Sciences, Shiraz, Iran

**Keywords:** Vitamin D deficiency, Testis, Testosterone, Rats, Reproduction, Stereology

## Abstract

**Background:**

Accumulating evidence suggests that low vitamin D status may affect male gonadal structure. This study was undertaken to reveal whether vitamin D-deficient rats have demonstrable changes in the quantitative histomorphometric properties of the testis.

**Methods:**

In the present investigation, adult male Sprague-Dawley rats were divided into four groups and received: group 1) conventional diet; group 2) vitamin D-deficient diet; group 3) vitamin D-deficient diet and paricalcitol and group 4) conventional diet plus paricalcitol. After 3 months, serum levels of vitamin D metabolites, Ca, P, LH, FSH, testosterone, and epididymal sperm quality were evaluated. Moreover, the morphometric characteristics of testis were assessed via stereological methods.

**Results:**

Rats fed a vitamin D-deficient diet (groups 2 and 3) were normocalcemic and had 25-hydroxyvitamin D_3_ level below 10 ng/mL. A significant reduction in serum testosterone and comparable gonadotropin levels were seen in vitamin D-deficient groups compared to controls. The concentration, morphology, and motility of sperm cells were profoundly disturbed in animals raised on the vitamin D-deficient diet. There was a significant decline in the population of different germ cells, the volume of interstitial tissue and germinal epithelium in group 2 and 3 rats, which were placed on the vitamin D-deficient diet. No appreciable difference in the estimates of the Leydig or Sertoli cell numbers were observed between groups.

**Conclusions:**

The depletion of vitamin D stores and induction of moderate grades of vitamin D deficiency by dietary measures led to remarkable impairment of spermatogenesis and microscopic architecture of rat testis. These findings can be attributed, at least in part, to decreased androgen production.

## Background

An estimated 15% of couples are affected by infertility globally. Identifiable male-related factors account for approximately 20–30% of infertility cases and contribute to 50% of reproductive problems overall [1]. However, there is no distinguishable cause in 30–45% of infertile men with dysspermatogenesis [2].

Vitamin D (VD) is a prohormone necessary for human health. The most active form, 1,25-dihydroxyvitamin D [1,25(OH)_2_D_3_] is a pleiotropic steroid hormone regulating calcium homeostasis and a wide variety of other biologic systems. In the context of the non-classical effects of VD in the biology of mammalian species, its impact on reproductive organs has received more attention in recent decades. Indeed, the established presence of vitamin D receptor (VDR) and VD metabolizing enzymes in male genital tract [3, 4] prompted several investigators to search for the relation between low vitamin D status as a common problem [5] and various kinds of male reproductive function including androgen production and spermatogenesis [6, 7].

Meanwhile, a general agreement about the exact role of VD in male fertility is still a subject of debate [6, 8]. Moreover, hypocalcemia and changes in other regulatory factors of calcium homeostasis such as parathyroid hormone often accompany VD deficiency and are a real barrier for proper interpretation of VD mediated effects [9, 10].

The aim of this study was to investigate the influence of experimental VD deficiency on rat male reproductive activities. For this purpose, we used stereological assessment of testis and evaluated varied parameters of sperm quantity and quality. In addition, we utilized a method for inducing VD depletion without concomitant disturbance in calcium homeostasis [8, 11–15] to elucidate the possible direct role of VD deficiency in the regulation of different aspects of rat male reproductive system.

## Method

The local ethics committee of Shiraz University of Medical Sciences approved the study. The experiment was conducted in accordance with the ARRIVE (Animal Research: Reporting of in Vivo Experiments) guidelines [16] for the care and use of research animals.

A total of 40 adult male Sprague-Dawley rats (10 weeks old), weighing 300 ± 25 g, were purchased from the animal laboratory center of Shiraz University of Medical Sciences. Subjects underwent one-week acclimatization to the animal laboratory facilities before the study. They were housed in standard laboratory conditions (20 ± 2 °C, 12,12 h light/dark cycle) with free access to food and water. After 1 week, they were simple randomly divided into four groups of 10 rats as follows:

A) Group 1 (control group) received a standard rat chow diet (SD) containing 10 g Ca and 7 g P per kilogram.

B) Group 2 received a customized vitamin D-deficient diet (VDD) [Royan laboratory animal feed, Isfahan, Iran] containing 20% lactose, 2% Ca, and 1.25% P.

C) Group 3 (VDD + P) were fed the same diet as group two. In order to accelerate the depletion of endogenous stores of active VD [15], they also received intraperitoneal injections of 32 ng of paricalcitol (Zemplar®), a synthetic vitamin D analogue [17], on days 1, 3, 5, 8, 10, and 12. Paricalcitol is a selective VDR activator that inhibits PTH synthesis and secretion with less hypercalcemic and hyperphosphatemic effects than calcitriol [17]. The stimulation of VDR by paricalcitol also induce the expression of CYP24A1 or vitamin D 24-hydroxylase, the major enzyme responsible for the degradation of active hydroxylated metabolites of VD, promoting depletion of bodily stores of these active forms [15].

D) Group 4 (SD + P) received the same diet as the control group plus intraperitoneal injections of paricalcitol as described in group 3.

Rats and food consumption were weighed weekly during the experiment. After 3 months, their weights were recorded before they were killed by an overdose of thiopental (100 mg/kg) under ketamine-xylazine (Alfasan, Netherland) anesthesia. Samples for semen analysis were obtained from the cut edge of left caudate epididymis [18]. Left testes were excised for weighing and stereological studies.

### Serum sampling and assay

After withholding food and water for 12 h, blood samples were taken from the studied rats by cardiac puncture at the end of the study. Serum total calcium was measured by spectrophotometric method using Arsenazo III by an automated chemistry analyzer. Serum phosphate was determined by spectrophotometer using ammonium molybdate. The level of 1,25(OH)_2_D_3_ (pg/mL) was assessed by enzyme-linked immunosorbent assay (catalog No: ZB-11556C-H9648, ZellBio GmbH, Germany) with intra-assay coefficient of variation (CV) less than 10%, inter-assay CV less than 12%, and lower detection limit of 1 pg/mL. 25-hydroxyvitamin D_3_ (25OHD_3_) was quantified using ElectroChemiLuminescence (ECL) method by means of cobas e 411 analyzer (Reference number: 07464215 190, Roche Diagnostics, GmbH, Mannheim, Germany) and lower detection limit of 3 ng/mL. The CVs were less than 7% for intra-assay variability and less than 10.5% for inter-assay variability.

The levels of gonadotropins (LH and FSH) and total testosterone were evaluated by immunoradiometric assay (IRMA) and radioimmunoassay (RIA) methods, respectively (product code: RK-750CT for LH, RK-790CT for FSH and RK-61CT for testosterone, Izotop, Budapest, Hungary). The lower detection limit for testosterone assay was 0.04 ng/ml.

### Sperm collection and analysis

Epididymal sperm were removed from the cut edge of caudal epididymis, washed and suspended in phosphate buffered saline. All samples were counted in a hemocytometer. Then, 10 fields were randomly selected and evaluated for grading motility to distinguish the immotile sperms from those with progressive or non-progressive motility [19]. Furthermore, the sperm smears were stained with 1% eosin Y to assess their morphology [19].

#### Stereological study

The previously described principles and methods were adopted for stereological evaluation of testis [20]. Briefly, left testis was immersed in isotonic saline-filled jar for estimating its weight and primary volume [21] and then fixed in a buffered formaldehyde solution. Afterwards, each testis was divided into 8–12 slabs using the orientator method to obtain Isotropic Uniform Random (IUR) sections [22]. Paraffin-embedded tissue specimens were cut into 5-μm-thick sections employed for volume estimation of specified items and 25-μm-thick sections used for evaluating cell numbers. The 5 and 25-μm-thick section series were stained with hematoxylin-eosin and Masson’s trichrome method, respectively.

### Estimating the degree of shrinkage and the total volume of testis

To minimize the effect of tissue processing and ensuing tissue shrinkage on estimating the true volume of testis, three random segments from the IUR sections were prepared by means of a trocar. Two vertical diameters of each segment were measured and their mean was considered as pre-fixing radius (r-before). Likewise, post-fixing radius (r-after) was calculated after histological processing. The mean degree of global tissue shrinkage [d (shr)] was calculated by [20]:

d (shr) = 1 - (r-before^2^ / r-after^2^)^1.5^.

The total (post embedding) volume of the whole testis was determined by the following formula, using the primary (pre-embedding) volume estimated by immersion method:

V (post-embedding) = V (pre-embedding) × [1 − d (shr)].

### Estimation of the volume densities and the total (absolute) volumes of seminiferous tubules, interstitial tissue, and germinal epithelium

The volume density was estimated using the point counting method. In brief, the eye piece graticule of the microscope was aligned to completely cover individual 5-μm sections from each testis. At a final magnification of × 280, images were captured and transferred to a computer screen for analysis by morphometric software (Stereo-Lite, Shiraz, Iran). An average of five fields per each 5-μm section was evaluated by randomly superimposing a uniform point grid over pictures to measure specified objects. The data generated from all sampled sections, fields, and counts were used for final calculations. To estimate the volumes of seminiferous tubules, interstitial tissue, and germinal epithelium, the following equation was applied:

Vv (structure) = ΣP (structure) / ΣP (reference).

Where the “ΣP_Structure_” is the number of points hitting the profiles of the tubules, interstitial tissue, or germinal epithelium, and “ΣP _reference_” is the number of points hitting the testis. Finally, the total volume of each compartment was calculated by multiplying its volume density (Vv) by the total volume of the testis [20].

### Stereological analysis of germ cell lineage, Leydig and Sertoli cells

Optical disector method and unbiased computer-generated frames were employed for counting all nuclei and final estimation of the total number of individual cells in each testis [23]. The procedure was done with a Nikon E200 light microscope (Tokyo, Japan) fitted with a 60x oil objective lens at a final magnification of × 1640 and a microcator (MT12, Heidenhain, Traunreut, Germany) used to monitor field depth. The numerical density (Nv) of the cells was calculated as follows:

Nv (cells) = [ΣQ / (ΣP × (*a/f*) × h)] × (t/BA).

Where ΣQ is the number of each cell type nuclei, *a/f* is the area per counting frame, ΣP is the total number of counted frames per animal; h is the height of the optical disector measured using the microcator, *t* is the mean thickness of final sections, and BA is the microtome block advance. The total number of favored cells per testis [N (cells)] were estimated by multiplying the numerical density (Nv) by the reference volume:

N (cells) = NV (cells) × V (structure).

Where V (structure) is the total volume of germinal epithelium for the cells in the germinal layer, and the total volume of interstitial tissue for Leydig cells.

### Statistical analysis

All variables are expressed as the mean ± SD. The differences between groups were tested statistically with the Kruskal-Wallis followed by the Mann-Whitney U test for pairwise comparisons. All data were analyzed using SPSS software (version 23; SPSS, Chicago, IL, USA), and the graphs were designed by Graph Pad Prism 5 (San Diego, CA, USA). A *P* value < 0.05 was considered to indicate statistical significance.

## Results

The results of all biochemical analyses are presented in Table 1. Serum Ca and P levels were similar in all groups. The mean 25OHD_3_ concentration decreased to < 10 ng/mL in rats treated with VDD and declined even further when a short course of paricalcitol was added to the VDD in group 3 rats at the beginning of the study. The 25OHD_3_ was significantly lower in group 4 than in the control group although it was higher than the observed results in groups 2 and 3. Concomitantly, 1,25(OH)_2_D_3_ was diminished significantly in rats provided with VDD compared to controls but it did not differ between those groups given VDD (groups 3 and 4) regardless of whether they received paricalcitol or not. Paricalcitol per se did not lower serum levels of 1,25(OH)_2_D_3_ compared to control animals.

**Table 1:** Results of serum biochemical investigations in the studied rats at 12 weeks^a^

***Group*** (n)	Ca (mg/dL)	P (mg/dL)	25OHD_**3**_ (ng/mL)	1,25(OH)_**2**_D_**3**_ (pg/mL)	LH (mIU/mL)	FSH (mIU/mL)	Testosterone (ng/mL)
**1 (Standard diet)**	9.8 ± 0.6	5.8 ± 0.8	32.9 ± 4.7^a^	25.6 ± 5.6^a^	0.16 ± 0.05	1.12 ± 2.7	1.96 ± 0.6^ac^
**2 (VDD)**^b^	9.5 ± 0.2	6.2 ± 0.6	7.6 ± 1.9^c**^	17 ± 7.3^b*^	0.46 ± 0.3	3.2 ± 4.5	0.19 ± 0.06^b*^
**3 (VDD + Paricalcitol)**	9.6 ± 0.7	6 ± 0.6	4.6 ± 2.3^d**^	14 ± 4^b**^	0.34 ± 0.2	1.04 ± 2.4	0.15 ± 0.04^b*^
**4 (Standard diet + Paricalcitol)**	9.7 ± 0.9	6 ± 1.2	19.7 ± 4.5^b*^	19.6 ± 7.5^ab^	0.41 ± 0.3	0.19 ± 0.1	1.2 ± 0.6^bc^

Means followed by a common small letter superscript are not significantly different and means followed by a different small letter superscript are significantly different, * < 0.005, as compared to group 1 (controls), ** < 0.0005, as compared to group 1 (controls)

^a^Values in the table are means ± SD, *n* = 9 per group

^b^Vitamin-D deficient diet

Assessment of gonadotropins showed no statistically significant difference in serum LH or FSH levels between groups. The testosterone concentration of the rats given VDD were lower than in the vitamin D-replete controls irrespective of receiving paricalcitol. It even reached the lower detection limit of the assay. Paricalcitol injection did not significantly change testosterone level on its own.

At 12 weeks, vitamin D deficient diet alone or combined with paricalcitol injection led to a significantly less weight gain than the control group and those who received paricalcitol in addition to the standard diet; with no difference between group 1 and 4. The rats assigned to the experimental groups had lower testis weight and absolute volume than in controls at the end of this study (Table 2), while no difference was observed between experimental groups regarding these entities. However, relative testis weight (mean testis weight/ mean body weight at the end of study period ×100) was not different between groups (Table 2).

**Table 2:** Stereological profile and estimation of different cell types (× 10^6^) of testis parenchyma in the control and experimental rats

***Group*** (n)	Relative Testis weight^**a**^ (%)	Testis volume (mm^3^)	Leydig Cells	Sertoli Cells	Total spermatogonia^b^	Round spermatid	Elongated spermatid	Spermatocyte
**1** (8)	0.48 ± 0.01	1.7 ± 1^a^	25 ± 5.5	36 ± 17.6	177 ± 75^ac^	250 ± 57^a^	361 ± 80^a^	136 ± 55^ac^
**2** (7)	0.5 ± 0.1	1.5 ± 0.3^b^	18.6 ± 7	31 ± 9.4	126 ± 43^bc^	111 ± 39^b^**	167 ± 73^b^**	80 ± 36^b^*
**3** (8)	0.49 ± 0.1	1.5 ± 0.3^b^	17.8 ± 7.5	28 ± 7.8	85 ± 38^b^*	101 ± 42^b^**	157 ± 72^b^**	79 ± 28^b^**
**4** (7)	0.47 ± 0.08	1.5 ± 0.2^b^	18.5 ± 6.8	33 ± 10	136 ± 60^bc^	210 ± 46^a^	219 ± 70^b^	102 ± 31^bc^

Values in the table are means ± SD, *n* = Numbers of animals in each group

Means followed by a common small letter superscript are not significantly different and means followed by a different small letter superscript are significantly different, * < 0.05, as compared to group 1 (controls), ** < 0.001, as compared to group 1 (controls)

^a^Relative Testis Weight = Mean testis Wt / Mean body Wt at the end of study period

^b^The mean of spermatogonia type A and B

Stereological quantitation of different cell populations of testis is depicted in Table 2. Sertoli and Leydig cell numbers were similar between experimental groups and controls. Spermatocyte and round spermatid count reduced significantly in rats fed VDD (groups 2 and 3), but paricalcitol did not affect these numbers by itself. The decline in elongated spermatid count was significant in rats fed VDD (groups 2 and 3) and those which received paricalcitol in addition to the standard diet (group 4). The mean total spermatogonia (spermatogonia A and B) count dropped by 52% in rats exposed to combined VDD and paricalcitol compared to their counterparts in the control group. The respective numbers of total spermatogonia count were similar between group 2 rats fed VDD and those on the vitamin D-sufficient diet (groups 1 and 4).

**Fig. 1:**
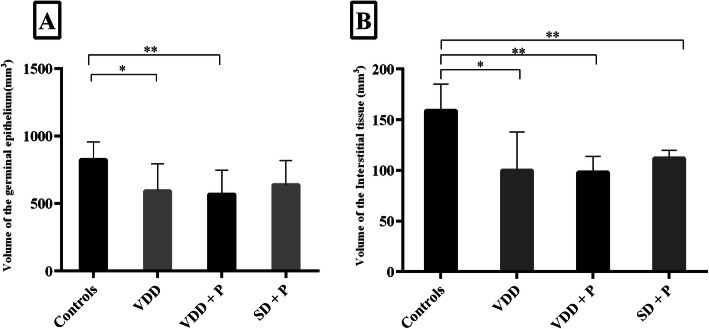
The effect of vitamin D-deficient diet (VDD, group 2), vitamin D-deficient diet and paricalcitol (VDD + P, group 3), and paricalcitol (standard diet+P, group 4) on the volume of germinal epithelium (**a**) and interstitial tissue (**b**). ^*^ *P < 0.05 and* ^**^P < 0.01, difference from controls (group 1)

Figure 1 illustrates the comparison of tubular germinal epithelium volume and interstitial tissue volume between the groups. The volume of interstitial tissue and seminiferous germinal epithelium decreased significantly in groups raised on a vitamin D-deficient diet (groups 2 and 3). A short course of paricalcitol injections in rats fed SD (group 4) was also accompanied by a reduction in the interstitial tissue volume with no effect on the germinal epithelium volume. No remarkable changes in tubular lumen volume were found between groups (data not shown).

Table 3 represents differences between the control and the experimental rats with respect to sperm quality and quantity parameters. Epididymal sperm concentration was reduced in rats offered low vitamin D diet, whether they were given paricalcitol or not. SD plus paricalcitol also led to a lower sperm count (group 4), but it was not different from the values seen in group 2. Quantitation of rapid progressive motility (RPM) of sperm (the percentage of sperms exhibiting rapid, linear movement), in the rats exposed to VDD revealed a significant drop during the study period. The paricalcitol exposure also led to lower percentages of RPM in the animals fed either VDD or standard diet. Conversely, the estimation of non-progressive forms of spermatozoa demonstrated higher values in the experimental groups.

**Table 3:** The values of varied epididymal sperm parameters in the control and experimental rats

Sperm motility (%)							
***Group*** (n)	Sperm count (× 10^6^)	Abnormal shape (%)	Viable sperm (%)	Immotile	Non-progressive	Slow	RPM^a^
**1** (8)	11.2 ± 2.3^a^	6.5 ± 2.1^a^	93.1 ± 4.8^a^	14 ± 5^b^	23 ± 8^a^	4 ± 3	58 **±** 12^a^
**2** (8)	5.7 ± 2.1^b^**	9.3 ± 1.9^b^*	76.5 ± 8.8^b^**	31 ± 25^ac^*	40 ± 15^b^*	10 ± 6	23 **±** 12^b^**
**3** (8)	3.2 ± 1.9^c^**	9.3 ± 0.9^b^*	73.1 ± 7.5^b^**	40 ± 13^a^**	41 ± 10^b^*	13 ± 8	7 **±** 5.6^c^**
**4** (8)	6.8 ± 4.2^bc^*	9.7 ± 1.9^b^*	72.8 ± 7.1^b^**	16 ± 6^bc^	43 ± 16^b^*	12 ± 6	28 **±** 17^b^*

Values in the table are means ± SD, *n* = Numbers of animals in each group

Means followed by a common small letter superscript are not significantly different and means followed by a different small letter superscript are significantly different, * < 0.05, as compared to group 1 (controls), ** < 0.005, as compared to group 1 (controls)

^a^Percentage of sperms showing rapid progressive movement (RPM)

## Discussion

Spermatogenesis is a multistep process regulated by complex interplay of endocrine, paracrine, and autocrine factors in addition to proper function of Sertoli and Leydig cells. Active VD metabolites modulate a wide range of genes throughout the body [24], but whether they are necessary for male reproductive function is far from clear [6, 25, 26]. In this comparative stereological evaluation of testis in the vitamin D-deficient Sprague Dawley rats, a considerable decline in germ cell populations accompanied by severe impairment of spermatogenesis and reduced androgen production was found. We attempted to find whether VD deficiency per se had a direct role in dysregulation of male gonadal performance in this study or the calcium deficiency secondary to lack of VD might be a causative factor [8, 11, 27, 28]. As normocalcemia was detected despite observing VD deficiency at the end of the present investigation, our study may serve as a model for exploring the possible direct adverse effects of moderate to severe VD deficiency on male gametogenesis in rats.

We used stereological examination of rat testis as a valuable tool for three-dimensional evaluation of volume of the germinal epithelium, germ cells in varying stages of differentiation, and Sertoli cells as the main elements of seminiferous tubules. This study demonstrated that proliferation of different populations of spermatogenic lineages was profoundly affected by VD deficiency. Consistently, the volume of germinal epithelium was also significantly decreased in the VD-deficient rats. Furthermore, the results showed the severe deficiency of 25OHVD_3_, as noted in group 3 rats, was associated with disturbed early stages of spermatogenesis marked by significant reduction of spermatogonia in this group compared to others.

Sertoli cells are essential for germ cell development during adult life [29]. Several studies [30, 31] have pinpointed Sertoli cells as a VD target of male rodent gonad. No difference in the number of Sertoli cells was observed between controls and cases in this study. Moreover, the serum levels of FSH as an indirect indicator of Sertoli cell function were not different between the groups. Androgen receptors are present within Sertoli cells, but germ cells do not express them [29, 32]. This fact suggests that the well-known role of testosterone in regulating spermatogenesis is mediated through Sertoli cells on the seminiferous epithelium [29, 32]. Hence, it seems that the observed reduction of germ cell population despite the undetected difference between the groups in the Sertoli cell number may be related to the diminished testosterone production (Table 1) in rats raised on VDD. Testosterone is particularly involved in providing support for spermatidogenesis [32]. Therefore, remarkable loss of round and elongated spermatids in the rats consuming VDD (56 and 54%, respectively) as opposed to the control group may further support the presumed role of androgen reduction in suppressing germ cells development during our present work. Leydig cells are responsible for androgen synthesis and are controlled by pulsatile LH secretion. Declined testosterone levels in rats maintained on a VD deficient-diet were not accompanied by a significant decrease in the Leydig cell population, although a non-significant tendency towards lower Leydig cell number was seen in the experimental groups exposed to VDD. Insufficient sample size may account for this unexpected result. Alternatively, it is plausible that the malfunction of the Leydig cells is responsible for the diminished androgen genesis in the VDD treated rats. Primary hypogonadism is characterized by corresponding LH rise secondary to reduced androgen synthesis. A probable explanation for unaltered LH concentration in response to low testosterone in rats placed on VDD; is pulsatile nature of the LH secretion and its short half-life. Errors may be introduced in measurements made on single samples [33]. The evidence of VDR expression in the pituitary gland has been shown by multiple methods [34]. Therefore, another possibility may be disturbed hormone secretion from pituitary cells after VD deficiency and development of secondary hypogonadism presenting with low or normal LH and low testosterone levels. But, an affirmation of these hypotheses in the present study was not possible.

The probable link between gonadal function and lack of VD effects has been investigated by several models [8, 25, 28, 35]. Published data regarding the relationship between sex hormone production and systemic changes of VD are controversial [25, 26, 30, 36]. Our experimental results are in agreement with some association and interventional studies in human [37, 38] and an animal study [39]. Hence, this issue needs to be explored in more detail to clarify unresolved discrepancies. Moreover, it is necessary to delineate the exact effect of vitamin D deficiency on gonadal and/or hypophyseal function in subsequent studies.

Another finding of the present experiment was disturbed epididymal sperm parameters related to semen quality. Sperm count, morphology, viability, motility, and the percentage of sperms showing RPM were profoundly affected by inducing VD deficiency. The relationship between the severity of VD deficiency and the extent of disturbance in the quality of sperm was more pronounced in group 3 rats, which were presented with the lowest 25OHVD_3_ levels. These data are consistent with and complement the histological abnormalities described above. Several animal studies have linked semen quality and specifically sperm motility to VD status [9]. A previous study found a similar suppression of spermatogenesis and histologic changes [11]. But, it occurred in the presence of hypocalcemia and completely resolved after correcting serum calcium with rescue diet. Contrary to our finding and others [11, 40], higher VD levels were associated with lower sperm count and percentage of sperms with normal morphology in a human study [41]. The reasons for these contradictory results regarding the exact effect of VD on spermatogenesis and the direct versus the indirect impact of VD on this process are not clear. Different species of mammals and strains of rodents which were investigated, and the dissimilarly achieved concentrations of 25OHD_3_ and 1,25(OH)_2_D_3_ may account for these apparent discrepancies to some extent. Collectively, it seems that in human subject’s substantial VD deficiency should be present to reproduce the same positive association between VD deficiency and disturbed sperm quality seen in animal studies. On the other hand, it has been reported that VD deficiency does not reduce 1,25(OH)_2_D_3,_ and therefore calcium absorption, until 25OHD_3_ falls to less than 2.5 ng/mL [42]. Accordingly, we postulate from our data that VD levels higher than amounts necessary for retarding the intestinal calcium absorption and reducing 1,25(OH)_2_D_3_ but lower than the defined normal range may be sufficient for interrupting normal gametogenesis and hormone production in male rat.

Some biologic systems like spermatogenesis take prolonged periods to occur. To better understand the influence of VD deficiency on these lengthy complex processes, the rapid achievement of VD-deficient state from the beginning of an animal study may aid the investigator. We employed a short course of paricalcitol injections in a group of rats fed with VDD (group 3) to accomplish this goal. Paricalcitol is a synthetic VD analogue [17], which upregulates 24 hydroxylation of VD metabolites via CYP24A1 with the subsequent inactivation and depletion of their bodily stores [15, 17]. It is noteworthy that the mean concentration of 25OHD_3_ reached the lowest level among the studied animals (< 5 ng/mL) in group 3 rats corresponding to the expected effects of paricalcitol. Although this degree of VD deficiency was not accompanied by the lower levels of 1,25(OH)_2_D_3_ or testosterone in comparison with the rats which received only a VD-deficient diet (group 2), a more pronounced reduction in sperm counts and percentage of sperms with RPM was seen. On the other hand, a less noticeable reduction of 25OHD_3_ was seen after the transient administration of paricalcitol to a group of rats given a standard diet (group 4). This raises the possibility that following the initial depletion of the endogenous VD by using this compound, the full recovery of VD stores did not occur throughout the study period. In any case, this mild form of VD deficiency in group 4 rats was not associated with declined testosterone levels. Nonetheless, a significant change in the quality of sperm parameters (Table 3) and disturbance in the terminal stages of spermatogenesis characterized by decreased elongated spermatids (Table 3), was detected. The analysis of these findings is important from a mechanistic point of view implying that the deteriorating effects of VD deficiency on spermatogenic process could not be fully explained by its reducing impact on androgen production. The possible role of aromatase and estrogen synthesis [7], osteocalcin [10], and calbindin-D28k [36] as the modulators of male gametogenesis has been proposed previously. A recent study concluded that a part of VD protective activity on gonadal function is exerted through peroxisome proliferator-activated receptor gamma/transforming growth factor beta 1/nuclear factor kappa B signaling pathway [43]. Further studies will be required to delineate the underlying mechanisms by which VD may exert direct or indirect regulatory function on the male reproductive tissue.

It is important to address the potential limitations of our study. We did not measure parathyroid hormone and FGF23 as the regulators of 1,25(OH)2D3 synthesis and degradation via CYP27B1 and CYP24A1. PTHrP protein and PTH/PTHrP receptor mRNA are present in the human and rat testis [44, 45]. Parathyroidectomy prevented the reduction of testosterone in dogs before inducing acute uremia [46]. Suppressive therapy of secondary hyperparathyroidism with VD analogues in adult ESRD patients restored the declined testosterone concentration in some [47] but not all [48] studies. Thus, the precise functions of PTH/PTHrP in the physiology of male gonad are unknown and remain to be determined. We could not exclude the contribution of the possible secondary hyperparathyroidism to the observed low testosterone levels in this VD-deficient animal model. However, the induction of VD-deficient state with a similar modified dietary regimen we employed here, was not associated with an elevated PTH level [13–15] even when the study period was extended up to 19 weeks [13]. One may argue that less body weight gain in VD-deficient animals throughout the present study may be a reason for the impaired spermatogenesis and hormone production. But in accordance with another experimental study [49], the relative testis weight was comparable in the studied groups (Table 2), and it does not seem to contribute to our observations significantly [28].

## Conclusion

Our results suggest that the experimental induction of VD deficiency by administering customized dietary regimen may give rise to significant impairment of male rat gametogenesis and histopathological abnormalities of testicular tissue. This is apparently due to the decreased androgen production secondary to moderate grades of VD deficiency. Nonetheless, the precise determination of the mechanisms by which VD deficiency exerts such deleterious effects on the gonadal function of male rat, and its relevance to human biology needs to be addressed by more investigations.

## Data Availability

All data generated and analyzed during this study are included in this article. The datasets used and/or analyzed during the current study are available from the corresponding author on reasonable request.

## Abbreviations

1,25(OH)_2_D_3_: 1,25-dihydroxyvitamin D
25OHD_3_: 25-hydroxyvitamin D_3_
VD: Vitamin D
VDR: Vitamin D receptor
VDD: Vitamin D-deficient diet
LH: Luteinizing Hormone
FSH: Follicle Stimulating Hormone
PTH: Parathyroid hormone
PTHrP: Parathyroid hormone-related protein
ARRIVE: Animal Research: Reporting of in Vivo Experiments
SD: Standard rat chow diet
VDD + P: Vitamin D-deficient diet+Paricalcitol
SD + P: Standard rat chow diet+ Paricalcitol
CV: Coefficient of Variation
ECL: Electrochemiluminescence
IRMA: Immunoradiometric assay
RIA: Radioimmunoassay
